
JOURNAL INFORMATION
==============================
NLM Title Abbreviation: Buffalo Med J Mon Rev Med Surg Sci
NLM Journal ID: 0004276

The Buffalo Medical Journal and Monthly Review of Medical and Surgical Science

Publisher: Biomedical Journal Digitization Project

ARTICLE INFORMATION
==============================
PMCID: PMC8932445
PMID: 35376942
Article version: 1
Subjects: Eclectic Department

Warning to Invalids Visiting the Island of Cuba

Print publication date: 1858 Dec
Volume: 14
Issue: 7
First page: 435
Last page: 436
License: This work is free of known copyright restrictions. For more information, please see PMC Back Issue Digitization.
License URL: https://creativecommons.org/publicdomain/mark/1.0/

==============================

mend to thoughtful perusal the valuable monograph on the subject of Anæsthesia which he has bequeathed to the profession. No one can rise from reading this valuable digest of a wide experience and the observation of ten years of scientific and practical labor, without a feeling of regret that so much carelessness should still prevail in the administration of this most potent vapor, and a sense of the necessity for a more extended instruction in the principles of anæsthetization. We commend the following sentences to the very careful consideration of surgeons:—

"The great point to be observed in causing insensibility by any narcotic vapor, is to present to the patient such a mixture of vapor and air as will produce its effects gradually, and enable the medical man to stop at the right moment. Insensibility is not caused so much by giving a dose as by performing a process. Nature supplies but one mixture of diluted oxygen, from which each creature draws as much as it requires; and so, in causing narcotism by inhalation, if a proper mixture of air and vapor is supplied, each patient will gradually inhale the requisite quantity of the latter to cause insensibility, according to his size and strength. It is, indeed, desirable to vary the proportions of vapor and air, but rather according to the purpose one has in view, whether medicinal, obstetric or surgical, than on account of the age or strength of the patient; for the respiratory process bears such a relation to the latter circumstances, as to cause each person to draw his own proper dose from a similar atmosphere in a suitable time.'

"The proportion of chloroform most suitable to produce insensibility, Dr. Snow found to be about four cubic inches of vapor, or rather more than five grains of chloroform to one hundred cubic inches of air. With a properly-arranged inhaler, it is easily possible to supply the vapor in this fixed position. This simple precaution would rob chloroform of nearly all its terrors and its dangers. It is a very surprising consideration, that while the niceties of surgical manipulation are invaribly attended to with the utmost care, the production of anæsthesia is very frequently left in the hands of quite inexperienced persons. It is sad to see the issues of life and death treated with indifference; it would be yet more so, if we were not well aware that the fault is one of thoughtlessness, and that those who have fallen into it will probably shrink from a similar error now that it has been pointed out so forcibly by recent events."—*Boston Med. and Surg. Jouraal.*

---

*Warning to Invalids Visiting the Island of Cuba.*—[We beg leave to present our readers the following extract from a letter received from an esteemed correspondent in Havana. It speaks for itself, and all medical men who send patients to Cuba for relief, should be careful to make every effort to secure them from the imposition pointed out.—Eds.]

If you like to take the trouble to give notice to your readers, that such of their patients and other invalids as come to Havana shall be counseled in respect to localities best suited to their conditions, or if so ill as to require private lodgings, or residences separate from the crowd and discomfort of hotels, it will give me much satisfaction to be of service to them in any way that may be in my power, and to carry out every judicious plan of treatment that may be recommended by their physicians.

I do not wish to be understood as advertising myself, or as soliciting cases, but the custom with infirmaries and several physicians, of employing runners and of paying commissions to catch strangers, renders it suitable that the profeasion be advised of the indecent usages and its objects; and the more so, that the parties guaranty quick and radical cures of diseases in the most desperate circumstances, stipulating beforehand the amount of money to be paid, usually from one to five thousand dollars, and the largest part in advance that can possibly be obtained. The patient is always required, under the pretext of air, diet, or something else, to take lodgings in some family in which the physician confides, and the members of which, and also their friends, are instructed in the part they are to act in the conspiracy. At the proper time, an interpreter is at hand; at other times, the patient is surrounded by persons he cannot understand or make understand him: the thieves soon seize his pile, and proceed in the process of fleecing their victim, whose effects in the end fall short of paying his bills; the flattering arrangement has cost the unfortunate patient his last dollar, with no result but a heartlsss robbery, and chagrin and bitter disappointment. Instances of this kind of financiering occur every season, and it happens, also, if the funds of the victim do not hold out to pay every dollar, and the regular course of the disease, or some other cause, has ended in the death of the patient, his acquaintances and countrymen must provide for his interment, or see his remains conveyed in the common dead-cart to the cemetery, and deposited without coffin or any covering in the ditch or common receptacle of the dead from hospitals, prisons, and places of execution. Strangers who are invalids, should consult their Consul, or some countryman of established character, in respect to whatever they propose to do, particularly in respect to medical attendants, and all matters involving important interests, with which they themselves are perfectly familiar. There is no need of any person falling into the hands of medical thieves and robbers. In Havana, there are great numbers of professional gentlemen, whose spotless honor and high attainments would lose nothing by any test or comparison, and whom money could not induce to guarantee the cure of a disease already advanced to the point which makes it necessarily mortal; but these do not employ runners or whippers-in, or divide their fees in commissions.

The credulity of invalids is notorious. Men of the best capacity, the highest attainments and stations, heads of the law and church, allow themselves to fall victims to the most grovelling impostures, and dupes of the vilest quackery. The best understandings are betrayed by credulity and fear, or importuned out of their senses by designing rogues, officious spectators, as well as by honest-meaning friends. If we consider how intensely life and health engage the hopes and fears of all, the blindest judgment in her vain groping for something to ward off despair, and her idiotic trust in ignorance and knavery ought rather to be matter of regret and commiseration, than of surprise and derision; and allowance should be made for the effects of sickness, for impatience from sufferings unrelieved, perhaps unrelievable by any skill.—*New Orleans Medical News and Hospital Gazette.*
